# Platelets convert peripheral blood circulating monocytes to regulatory cells via immunoglobulin G and activating-type Fcγ receptors

**DOI:** 10.1186/s12865-015-0086-z

**Published:** 2015-04-21

**Authors:** Masanori Inui, Kino Tazawa, Yoshiro Kishi, Toshiyuki Takai

**Affiliations:** Department of Experimental Immunology, Institute of Development, Aging and Cancer, Tohoku University, 4-1 Seiryo-machi, Aoba-ku, Sendai 980-8575 Japan; Medical & Biological Laboratories, Co., Ltd., 4-5-3, Sakae, Naka-ku, Nagoya 460-0008 Japan

**Keywords:** Regulatory response, IL-10, Platelet, Fc receptor, Monocyte

## Abstract

**Background:**

Monocytes and macrophages produce interleukin (IL)-10, an immunoregulatory cytokine and a potent therapeutic tool for immune disorders. Augmentation of IL-10 production with a concomitant reduction of proinflammatory cytokines in macrophages *in vitro* is attained by doubly stimulating the cells with a toll-like receptor ligand and immunoglobulin (Ig)G immune complexes, a response known as that of regulatory (or alternatively activated/M2) macrophages. However, it has not been explored sufficiently how such a regulatory response could be exploited for anti-inflammation. Our objective is to find a potential way or condition for augmenting IL-10 by monocytes/macrophages *in vivo* and *in vitro*.

**Results:**

We show that platelets, when they are opsonized with IgG, can convert human peripheral blood circulating monocytes to IL-10-producing regulatory monocytes *in vitro* and also in a murine *in vivo* model. Co-culturing of platelets and monocytes in the presence of anti-integrin IgG and a bacterial lipopolysaccharide augmented IL-10 production via a direct interaction between platelets and monocytes. This novel way of enhancing IL-10 was mediated by activating-type Fc receptors for IgG.

**Conclusion:**

These findings indicate that the IgG-bound platelet-induced conversion of monocytes to regulatory cells might provide a novel strategy for controlling inflammation.

**Electronic supplementary material:**

The online version of this article (doi:10.1186/s12865-015-0086-z) contains supplementary material, which is available to authorized users.

## Background

Monocytes in the bloodstream and macrophages in tissues play important roles in immunity not only as inflammatory initiators by releasing a series of proinflammatory cytokines and chemokines [1], but also as regulators of the inflammation by producing a potent anti-inflammatory or immunoregulatory cytokine, IL-10 [2-6]. Recently, the IL-10-mediated anti-inflammatory mechanism of monocytes/macrophages as well as of many other leukocytes such as regulatory T cells and IL-10-producing B cells has attracted much interest because of its potential therapeutic benefit for immune disorders including inflammation and autoimmune diseases. Therefore, it is important to find an efficient way to induce IL-10 from such leukocytes, particularly monocytes/macrophages, as a rich source of IL-10 in the bloodstream and tissues [7-13].

One prominent IL-10-producing mechanism of monocytes/macrophages *in vitro* is that of regulatory (or alternatively activated or M2) macrophages, i.e., cells stimulated with a TLR ligand such as LPS in conjunction with IgG-immune complexes (IgG-ICs) like those comprising OVA and anti-OVA IgG, or other stimuli including prostaglandins, G-protein-coupled receptor ligands, glucocorticoids, apoptotic cells, or IL-10 itself [14]. Signaling of the IL-10 up-regulation by LPS- and IgG-IC-stimulated macrophages has been revealed to involve ERK, p38, MAF, and NF-κB [10]. Also experimentally, murine macrophages are converted to regulatory cells via IgG-bound sheep erythrocytes [14,15] on simultaneous LPS stimulation. IgG-bound *Leishmania* exploits the regulatory-type activation of macrophages for its parasitic invasion into the macrophages, and permissive growth intracellularly in humans and mice [16]. However, it is not known how regulatory macrophages are induced in more physiologic settings.

During our testing of a series of IgG mAbs against human PBMCs as to their cytokine induction effects, we found that some mAbs can augment IL-10 production by PBMCs stimulated with LPS *in vitro*. Detailed analyses revealed that the targets of these IgG mAbs were integrins on platelets and monocytes coexisting in the PBMC preparations, and that these antibodies induced a direct interaction between platelets and monocytes through Fc receptors for IgG, FcγRs. In this article, we show a novel converting mechanism, in which IgG-opsonized platelets evoke a regulatory cascade of circulating monocytes via activating-type FcγRs and augment IL-10 production by the monocytes.

## Methods

### Reagents and Abs

Histopaque1077 for the purification of PBMCs was obtained from Sigma-Aldrich (St. Louis, MO). PE-labeled mouse anti-human CD14 (clone M5E2) was purchased from BD Biosciences (San Jose, CA). Allophycocyanin-labeled mouse anti-human CD16 (3G8), mouse anti-human CD41 (HIP8), mouse anti-human CD42b (HIP1), mouse anti-human CD9 (HI9a), and mouse anti-human CD36 (5-271) were obtained from BioLegend (San Diego, CA). Mouse anti-human CD32 (IV.3) was obtained from StemCell Technologies (Vancouver, Canada). F(ab’)_2_ of the anti-human CD41 and isotype control mAbs were prepared using a Mouse IgG1 Fab and F(ab’)_2_ Preparation Kit (Thermo Scientific, Rockford, IL) according to the manufacturer’s instructions. Other murine mAbs as to human platelet antigens including #33 anti-human CD61 IgG2a were generated by Medical & Biological Laboratories, Co., Ltd. (Nagoya, Japan). The characters of several mAbs are summarized in Additional file 1: Table S1.

### Cell sorting and preparation of human platelets

This study was approved by the Ethics Committee of the Tohoku University Graduate School of Medicine and performed in accordance with a statement on ethical principles for medical research involving human subjects made in the Declaration of Helsinki. Cryopreserved human PBMCs were obtained from Cellular Technology Ltd. (Shaker Heights, OH). Frozen PBMCs were rapidly thawed in a water bath at 37°C, washed and resuspended in RPMI 1640 medium containing 10% FCS. Human PBMCs and mouse bone marrow (BM) cells were subjected to immunofluorescence staining, and sort-purified with a FACSAria III (BD Biosciences). Sort-purified monocytes were isolated from PBMCs based on forward scatter and side scatter characteristics and CD14 expression. The data were analyzed with FACS Diva (BD Biosciences) and FlowJo (Tree Star, Inc., Ashland, OR) software.

For the isolation of platelets, peripheral blood from healthy donors was drawn into tubes containing acid citrate dextrose, and a crude platelet-rich plasma fraction was isolated from the whole blood by centrifugation at 230 × *g* for 15 min at room temperature (RT). For the purification of platelets, the crude platelet-rich plasma fraction was centrifuged at 1,600 × *g* for 15 min at RT, and the pelleted platelets were resuspended carefully in the original blood volume of RPMI 1640 medium containing 10% FCS. The purified platelet preparation contained about 1 × 10^5^ counts of platelets/μl. The concentration differed from one preparation to another mainly due to the various donors (data not shown), and we used the purified platelet preparations for experiments without further adjustment. For the preparation of paraformaldehyde (PFA)-fixed platelets, purified platelets were treated with 1% PFA for 10 min at RT, and then washed with PBS three times.

### Cell cultures and LPS stimulation

Human PBMCs and sort-purified monocytes suspended in RPMI 1640 medium supplemented with 10% FCS were seeded onto a 96-well round-bottom plate (Greiner Bio-one, San Diego, CA), and then stimulated with 1 μg/ml LPS or 100 μg/ml poly(I:C) for 6–48 h in the presence or absence of human platelets and anti-platelet mAbs. For the FcγR-blocking experiments, anti-human CD32 (IV.3) or anti-human CD16 (3G8) mAbs were added to the wells to a final concentration of 1 μg/ml. Mouse BM cells were collected from femur and tibia bones, and suspended in RPMI 1640 medium supplemented with 10% FCS. Mouse BM-derived macrophages were obtained by culturing BM cells for 6–7 days in the presence of 20 ng/ml recombinant mouse M-CSF (PeproTech, Rocky Hill, NJ). Mouse BM cells and BM-derived macrophages were seeded onto a 96-well round-bottom plate, and then stimulated with 0.01–1 μg/ml LPS for 24 h in the presence or absence of human platelets and anti-platelet mAbs.

### Transwell experiments

Human PBMCs or mouse BM cells were seeded at 2 × 10^5^ cells/well into the lower chamber of a 24-well plate (Greiner Bio-one). Human platelets were cultured in the lower chamber directly in contact with the target cells or in the upper chamber separated from the target cells by a 0.4-μm pore membrane (Greiner Bio-one), which allows diffusion of small molecules, such as cytokines, but not platelets. Cells were stimulated with LPS for 24 h in the presence of anti-platelet mAbs and the culture supernatants were collected for measurement of cytokine production.

### ELISA

ELISAs were performed to measure human IL-1β, human IL-10, human IL-12, mouse IL-6, mouse IL-10, and mouse IL-12 using Human IL-1β ELISA Ready-SET-Go!, Human IL-10 ELISA Ready-SET-Go!, Human IL-12 ELISA Ready-SET-Go! (eBioscience, San Diego, CA), Mouse IL-6 ELISA MAX standard, Mouse IL-10 ELISA MAX standard, and Mouse IL-12 ELISA MAX standard, respectively (BioLegend). All ELISAs were performed using 96-well half-area microplates (Greiner) according to the manufacturer's directions.

### Mice

C57BL/6 (B6) mice were purchased from Charles River (Tokyo, Japan) and CLEA Japan (Tokyo, Japan). *FcRγ*^−⁄−^ mice [17], *Fcgr2b*^−⁄−^ mice [18], and *Fcgr3*^−⁄−^ mice [19] were backcrossed to B6 mice for 16, 22, and 6 generations, respectively. Mice were maintained and bred in the Animal Facility of The Institute of Development, Aging and Cancer, Tohoku University, an environmentally controlled and specific pathogen-free facility, according to guidelines for experimental animals defined by the University, and animal protocols were reviewed and approved by the Animal Studies Committee of the University. All experiments were performed on 8- to 12-week-old male and female mice.

### LPS-induced peritonitis

Mice were injected intraperitoneally with LPS at 5 mg/kg body weight. Anti-CD61 mAb, N6E4, or the isotype-matched control was intravenously administered 1 h before LPS challenge. Mice were sacrificed 3 h after LPS treatment, and then serum was collected.

### Statistical analysis

Statistical analysis was performed using Microsoft Excel for Mac 2011 software version 14.2.3 (Microsoft Corp., Seattle, WA, USA). Data are displayed when appropriate as means ± SD. Data were compared for statistical differences using Welch's *t*-test. *P* values are shown in the relevant figures. *P* < 0.05 was considered as statistically significant.

## Results

### Platelet antibodies augment IL-10 release from peripheral blood monocytes

**Figure 1:**
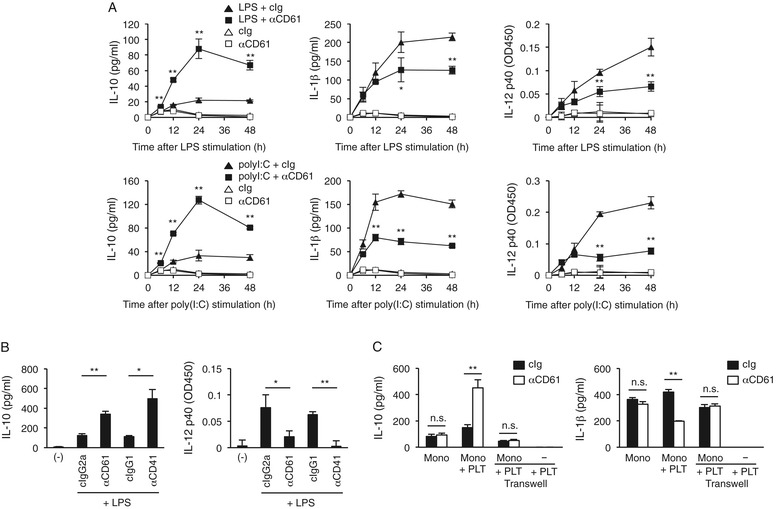
IgG-opsonized platelets augment IL-10 release but decrease proinflammatory cytokine release from monocytes via their direct contact. **(A)** A crude PBMC preparation was stimulated with LPS or poly(I:C) in the presence of anti-human CD61 mAb or isotype-matched control (cIg). The IL-10, IL-1β, and IL-12 p40 levels in the culture supernatants collected at different time points were determined by ELISA. **(B)** A crude PBMC preparation was stimulated with LPS in the presence of an anti-CD61 or anti-CD41 mAb or isotype control (cIg). The IL-10 and IL-12 levels in the culture supernatant after 24 h were determined by ELISA. Blank (−) indicates cells with no antibody or stimulator for monitoring spontaneous production of cytokines. **(C)** Transwell assaying of cytokine production. Sort-purified monocytes were stimulated with LPS in the presence or absence of platelets and an anti-CD61 mAb. The bottom chamber contained monocytes or was left blank, and the upper chamber contained platelets. Both chambers contained LPS and the anti-CD61 mAb. The IL-10 (*left*) and IL-1β (*right*) levels in the culture supernatant were measured after 24 h. *P < 0.05; **P < 0.01; n.s., not significant.

The addition of anti-human CD61 (integrin GPIIIa) (Additional file 1: Table S1) or anti-CD41 (GPIIb) monoclonal IgG to a PBMC preparation in the presence of LPS or poly(I:C) was found to be stimulatory as to the release of IL-10, but inhibitory as to production of IL-1β and IL-12 (Figure 1A,B). This cytokine modulation in monocytes was reminiscent of that in regulatory (or alternatively activated/M2) macrophages [1,14,15], in that macrophages were stimulated with a TLR ligand and IgG-ICs. In our assay system, the cytokine modulation required a direct interaction between the sorted monocytes and IgG-bound platelets, as verified on transwell assaying (Figure 1C). Monoclonal IgG molecules, which could avidly bind to platelet-surface antigens, such as CD9, CD36 (GPIIIb/IV), CD42b (GPIb) and CD226, were also able to augment IL-10 and to reduce IL-1β release as well (Additional file 1: Figure S1A–C). We concluded that the increased IL-10 release from monocytes is due to the direct association with IgG-bound platelets.

### IgG-bound platelet-induced regulatory response is mediated by FcγRII on monocytes

**Figure 2:**
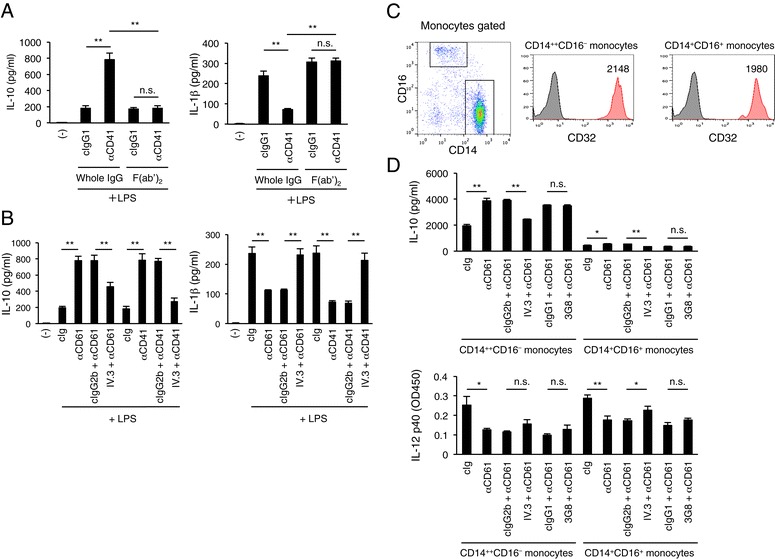
FcγRs mediate IgG-bound platelet-induced regulatory response in human peripheral blood circulating monocytes. **(A)** PBMCs were stimulated with LPS in the presence of whole IgG of an anti-CD41 mAb or an equimolar amount of the F(ab’)_2_ fragment, and then the production of IL-10 and IL-1β was measured after 24 h. Blank (−) indicates cells with no antibody or stimulator for monitoring spontaneous production of cytokines. Data are shown as means for triplicate samples ± SEM. The results are representative of more than three independent experiments with similar results. *P < 0.05; **P < 0.01; n.s., not significant. **(B)** FcγRII mediates IgG-bound platelet-induced regulatory response. PBMCs were incubated with an FcγRIIA/B-blocking mAb (IV.3), and then stimulated with LPS in the presence of an anti-CD61 or anti-CD41 mAb or its cIg. Data are shown as means for triplicate samples ± SEM. The results are representative of more than three independent experiments with similar results. *P < 0.05; **P < 0.01. **(C)** Robust FcγRIIA/B expression on circulating CD14^++^CD16^−^ and CD14^+^CD16^+^ monocytes. The monocytes were sort-purified from human PBMCs and tested for their CD32 (FcγRIIA/B) expression. **(D)** Sorted CD14^++^CD16^−^ and CD14^+^CD16^+^ monocytes were treated with an anti-CD61 mAb in the presence of CD32 (FcγRIIA/B)- or CD16 (FcγRIIIA/B)-blocking mAb, IV.3 or 3G8, respectively, or an isotype control, and then stimulated with LPS in the presence of human platelets. The IL-10 and IL-12 levels in the culture supernatants were measured after 24 h. Data are shown as means for triplicate samples ± SEM. The results are representative of more than three independent experiments with similar results. *P < 0.05; **P < 0.01; n.s., not significant.

We speculated that the IgG-bound platelet-induced regulatory response is mediated by Fc receptors for IgG (FcγRs) expressed on monocytes because they robustly express several types of FcγRs including activating type receptors FcγRIIA and FcγRIIIA as well as a unique inhibitory, FcγRIIB [20-22]. *Vice versa*, monocytes opsonized with IgG can also serve as a target for FcγRIIA uniquely expressed on human platelets. This notion was partly verified by our data shown in Figure 2A, in that the removal of the Fc portions of mAbs targeted to human CD41 attenuated the IgG-bound platelet-induced regulatory response. In addition, an FcγRIIA/B-blocking antibody, IV.3, attenuated IL-10 and restored IL-1β (Figure 2B), indicating that FcγRIIA/B on monocytes are involved in this response and that FcγRIIA on platelets could also play a role.

Human peripheral blood circulating monocytes are classified into two types, CD14^++^CD32(FcγRIIA/B)^+^CD16(FcγRIIIA)^−^ cells, which correspond to "classical monocytes" in mice, and CD14^+^CD32^+^CD16^+^ cells, which correspond to murine "non-classical or resident monocytes" [14,21,23,24], the former comprising the majority (80–90%) in human peripheral blood. Classical monocytes are those that infiltrate acute inflamed tissues in response to chemokines, whereas non-classical monocytes are assumed to infiltrate normal tissues and to contribute to homeostasis. With FcγR expression on these cells, it is expected that both the CD32^+^ classical and CD16^+^ non-classical type monocytic populations mediate the IgG-bound platelet-induced regulatory response, although a major mediator would be the classical ones due to their abundance in blood. To test this, we sort-purified the major CD14^++^CD16^−^ and minor CD14^+^CD16^+^ populations from a peripheral blood sample, confirmed their FcγRIIA/B expression (Figure 2C), and then measured the IgG-bound platelet-induced regulatory response activity of each monocytic population. We found that the classical monocytes mediated a much more robust IL-10 response than non-classical monocytes did, which was attenuated by IV.3, an FcγRIIA/B blocking mAb (Figure 2D *upper*). Down-modulation of IL-12 was evenly observed both in the classical and non-classical monocytes, which was also attenuated by IV.3 (Figure 2D *lower*). Blocking effect of the mAb 3G8, an FcγRIIIA/B blocker, was not evident. These results indicate that the classical monocytes in PBMCs and their FcγRIIA/B are the main mediators of IgG-bound platelet-induced regulatory response, albeit we do not rule out a contribution of non-classical monocytes and their FcγRIIIA.

### Pre-fixed platelets but not plastic microbeads can mediate regulatory response

**Figure 3:**
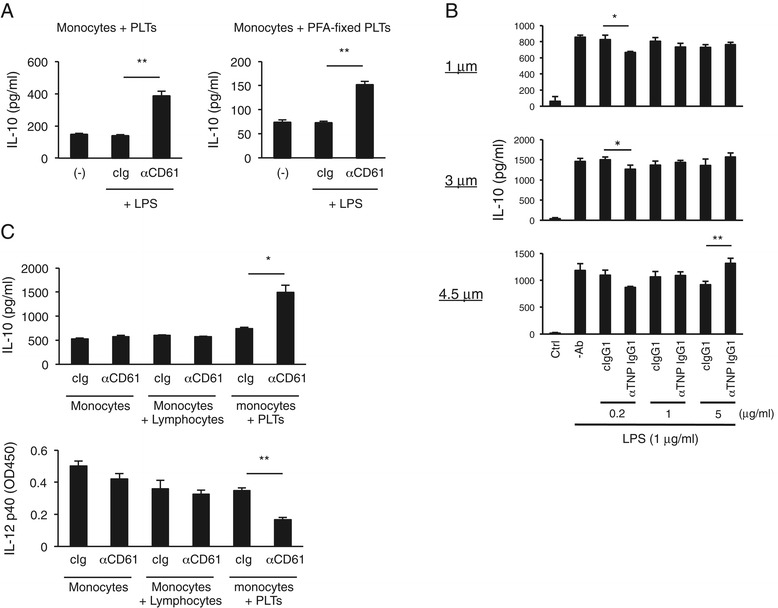
Pre-fixed platelets but not plastic microbead particles nor lymphocytes mediate regulatory response. **(A)** Pre-fixed platelets can mediate IgG-bound platelet-induced regulatory response. Paraformaldehyde (PFA)-fixed human platelets and sort-purified monocytes were incubated with an anti-CD61 mAb following LPS stimulation for 24 h. The IL-10 levels in culture supernatants were determined by ELISA. Data are shown as means for triplicate samples ± SEM. The results are representative of more than three independent experiments with similar results. **P < 0.01. **(B)** Plastic microparticles cannot mimic platelets. Anti-TNP-IgG-coated polystyrene microparticles of different diameters (1, 3, and 4.5 μm) were incubated with mouse bone marrow cells, which were then stimulated with LPS. After 24 h, the IL-10 levels in the supernatants were measured by ELISA. Data are shown as means for triplicate samples ± SEM. The results are representative of more than three independent experiments with similar results. *P < 0.05; **P < 0.01. **(C)** Sorted lymphocytes cannot mimic platelets. Sort-purified monocytes were subjected to the assaying with sorted lymphocytes or platelets as control in the presence of anti-CD61 mAb and LPS. The IL-10 and IL-12 levels in culture supernatants were determined by ELISA. Data are shown as means for triplicate samples ± SEM. The results are representative of more than three independent experiments with similar results. *P < 0.05; **P < 0.01.

Upon activation, platelets release the contents of their α-granules and up-regulate various cell-surface molecules such as P-selectin [25]. We wondered if platelet molecule(s) newly induced upon binding with anti-integrin IgG could be involved in the IgG-bound platelet-induced regulatory response, in addition to the constitutively expressed FcγRIIA and integrins. To examine this, we first fixed platelets with paraformaldehyde and then subjected them to the assaying. As shown in Figure 3A, the pre-fixed platelets mediated IL-10 augmentation, albeit the IL-10 level was lower than that induced by intact platelets, suggesting that molecules induced upon IgG binding, if any, are not indispensable for the response. We also wondered if plastic microbeads coated with IgG could mimic anti-integrin IgG-coated platelets. To examine this, we coated trinitrophenylated (TNP) polystyrene microbeads with anti-TNP IgG, whose robust binding to the beads was confirmed by flow cytometry (data not shown), and then subjected them to assaying. We found that IgG-coated microbeads of different diameters (1–4.5 μm) did not show a consistent IL-10 response (Figure 3B). We also tested if lymphocytes among PBMCs can mimic platelets because lymphocytes are weakly positive for integrin expression (Additional file 1: Figure S1A), and observed that these cells did not stimulate IL-10 or inhibit IL-12 release (Figure 3C). Collectively, these results suggest a specific role of platelets in the regulatory response, which cannot be played by plastic particles or lymphocytes, and the participation of unidentified surface molecule(s) other than integrins and FcγRIIA on platelets for fully effective response.

### Platelet-induced regulatory response is mediated by activating-type FcγRs also in mouse settings *in vitro*

**Figure 4:**
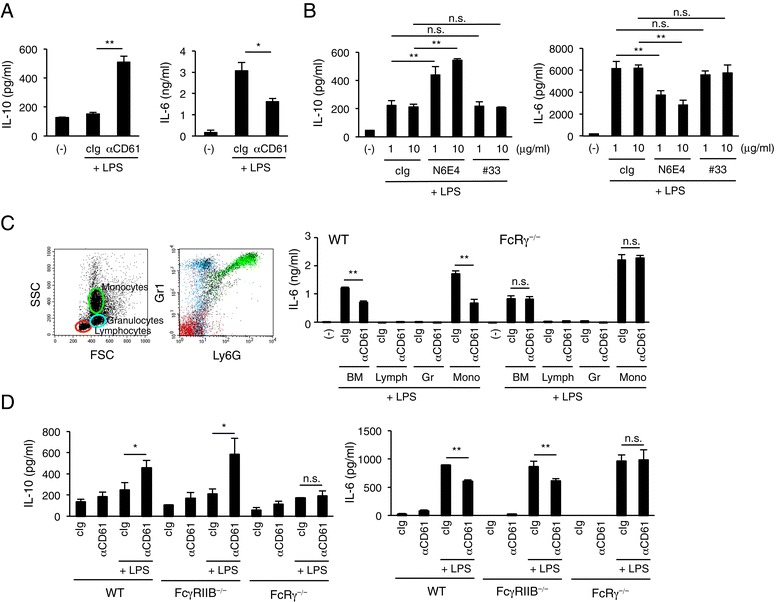
Activating-type FcγRs but not FcγRIIB mediates IgG-bound platelet-induced regulatory response in a mouse monocyte setting *in vitro*. **(A)** Mouse bone marrow (BM) cells were incubated with LPS in the presence of human platelets and an anti-human/mouse CD61 mAb (6E4), and then the production of IL-10 and IL-6 was assessed after 24 h. *P < 0.05; **P < 0.01. **(B)** Mouse BM cells were incubated with LPS in the presence of "mouse" platelets and anti-human/mouse CD61 mAb 6E4 or anti-human but not -mouse CD61 mAb #33 or cIg, and then the production of IL-10 (*left*) and IL-6 (*right*) was assessed after 24 h. #33 mAb did not cause the regulatory response in this all-mouse setting. **P < 0.01; n.s., not significant. **(C)** Neither granulocytes nor lymphocytes mediate regulatory response. Gr1^+^Ly6G^−^ monocytic, Gr1^+^Ly6G^+^ granulocytic and Gr1^−^Ly6G^−^ lymphoid cells were sorted from BM cells of wild-type B6 or FcRγ-deficient mice, and stimulated with LPS in the presence of "human" platelets and 6E4 or cIg. IL-6 was measured 24 h after stimulation by ELISA. **P < 0.01; n.s., not significant. **(D)** BM-derived cultured macrophages from wild-type, FcRγ-deficient or FcγRIIB-deficient mice were treated with anti-CD61 mAb 6E4 and stimulated with LPS in the presence of human platelets. The IL-10 and IL-6 levels in the culture supernatants were measured after 24 h. Blank (−) indicates cells with no antibody or stimulator for monitoring spontaneous production of cytokines. Data are shown as means for triplicate samples ± SEM. The results are representative of more than three independent experiments with similar results. *P < 0.05; n.s., not significant.

To confirm the involvement of FcγRs and other molecules *in vitro*, and to evaluate the *in vivo* effect of the IgG-bound platelet-induced regulatory response, we next attempted to construct a cross-species setting, in which human platelets interact with mouse leukocytes, and also an all-mouse setting. We incubated mouse bone marrow (BM) cells with human platelets in the presence of LPS and a mouse IgG2a mAb (6E4), which is reactive to both human and mouse CD61 (Additional file 1: Table S1). We found this human platelet–mouse BM cell setting acted as the IgG-bound platelet-induced regulatory response in terms of augmentation or inhibition of mouse IL-10 or IL-6, respectively (Figure 4A). In addition, culturing of mouse BM cells with "mouse" platelets in the presence of LPS and with 6E4 resulted in the regulatory response, but not with a mAb (#33) against human CD61, which does not bind to mouse CD61 (Figure 4B, see Additional file 1: Table S1), indicating that the IgG-bound platelet-induced regulatory response occurs in cross-species and mouse–mouse settings *in vitro* as well if we choose a suitable anti-mouse platelet antibody.

In the human–mouse setting, as shown in Figure 4C, Ly6G^−^ monocytic cells but not lymphocytes or Ly6G^+^ granulocytes among mouse BM cells exhibited the response in terms of suppression of IL-6 release, and the cells from FcR common γ subunit (FcRγ)-deficient mice had lost the ability of the response, suggesting that FcRγ-associating, activating-type FcγRs are involved. Mouse BM-derived cultured macrophages also showed the IgG-bound platelet-induced regulatory response, and again FcRγ was necessary but a unique inhibitory FcγR, FcγRIIB, was found to be dispensable for this effect (Figure 4D). Our additional examinations revealed that FcRγ-associating FcγRIII is involved in the regulatory response when a murine IgG1 anti-human platelet antibody was employed (Additional file 1: Figure S2), while an IgG2a antibody still induced the response even in the absence of FcγRIII, suggesting other activating FcγRs, such as FcγRI and FcγRIV, are also involved. Collectively, in the mouse setting *in vitro*, the IgG-bound platelet-induced regulatory response requires activating-type FcγRs but not FcγRIIB.

### Platelet-induced regulatory response works in a mouse *in vivo* model

**Figure 5:**
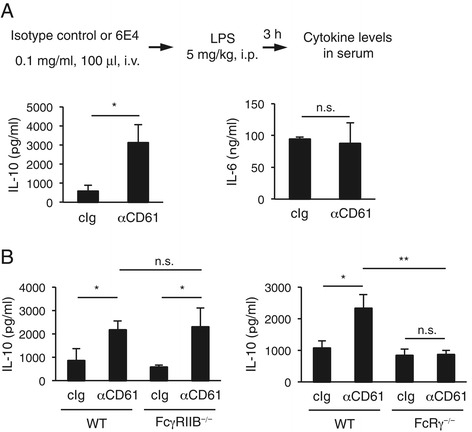
Augmented serum IL-10 in a murine *in vivo* setting. **(A, B)** Wild-type B6 mice were administered intravenously an anti-human/mouse CD61 or isotype control mAb and subsequently with LPS intraperitoneally. The IL-10 and IL-6 levels in serum samples collected at 3 h after LPS stimulation were measured by ELISA **(A)**. IL-10 upregulation in sera was observed also in FcγRIIB-deficient but not FcRγ-deficient mice (*n* = 4 mice per group) **(B)**. The results are representative of more than three independent experiments with similar results. *P < 0.05; **P < 0.01; n.s., not significant.

We next examined if IgG-bound platelet-induced regulatory response occurs in *in vivo* settings. We determined the cytokine levels in serum of a systemic septic shock model, in which mice were administered intravenously an anti-CD61 mAb and subsequently a sublethal dose of LPS intraperitoneally. As shown in Figure 5A, we observed that IL-10 was up-regulated while IL-6 was not affected, compared to in mice that received isotype-matched control antibodies, suggesting that IgG-bound platelet-induced regulatory response occurs, at least partly, in terms of IL-10 up-regulation. We verified again in this *in vivo* model that FcRγ-associating FcγRs but not FcγRIIB play a significant role in the response (Figure 5B). We do not know why a proinflammatory cytokine, IL-6, did not decrease in this *in vivo* setting, but additional IL-6-producing cells other than monocytes at the periphery might be involved.

## Discussion

**Figure 6:**
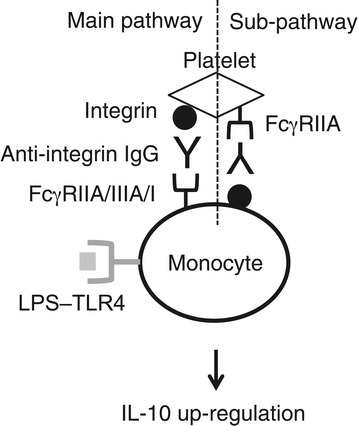
A proposed scheme for IgG-bound platelet-induced regulatory response. In IgG-bound platelet-induced regulation, a reciprocal interaction of IgG-opsonized platelets with monocytes via FcγRs constitutes the main and sub pathways. The main pathway is mediated by platelet surface molecules such as integrins, and IgG and activating-type FcγRs including FcγRIIA/IIIA/I on monocytes. The sub pathway includes monocyte-surface molecules such as integrins, and IgG and FcγRIIA on platelets. We do not rule out the potential involvement of a platelet-surface molecule(s), which contributes to the interaction with monocytes. Since platelets are much smaller than monocytes, it is likely that these pathways for a monocyte will be attained with multiple platelets.

In this study, we described a novel, IL-10 inducing, switching mechanism converting peripherally circulating monocytes to regulatory cells, involving IgG-bound platelets and activating-type FcγR (Figure 6). Platelets are generally known as a key player in blood clotting and wound repair processes, namely, the orthodox role of platelets in platelet-rich plasma or PRP therapy. In addition, platelets are an important accelerator of inflammation by releasing proinflammatory cytokines and chemokines, as well as by binding to various effector leukocytes including monocytes and neutrophils [1,3,26]. In an autoimmune arthritis animal model, for example, it has been demonstrated that platelets can amplify inflammation by producing microparticles derived from their own, which bind to activated polymorphonuclear leukocytes, thereby eliciting cytokine responses of synovial fibroblasts via interleukin IL-1 [27]. This binding process is dependent on glycoprotein IV or GPIV on platelets, and on the microparticles and collagen on inflammatory leukocytes [27]. Also, platelets activated by thrombin or other agonists can bind to monocytes in a P-selectin-dependent manner thereby inducing the production of proinflammatory cytokines by those cells. Importantly, however, when platelets are once activated, subsequent inflammatory responses induced by them should be adequately suppressed so as not to evoke excessive inflammation harmful for tissues. One mechanism for regulation of such platelet-initiated inflammation will be engulfment and degradation of the platelets by monocytes/macrophages. In this study, we found that platelets have a potential to convert activated monocytes/macrophages to regulatory cells when they are complexed with anti-platelet IgG molecules, which could be another mechanism for regulation of platelet-initiated inflammation.

Regulatory (or alternatively activated/M2) macrophages [15,23,24,28] are induced *in vitro* or in some infection models *in vivo* by small ICs such as OVA and anti-OVA antibodies [29], or particulate ICs such as IgG-opsonized sheep erythrocytes [14] or IgG-bound pathogen *Leishmania major* [16], in addition to by TLR stimulation with ligands such as LPS or live *Listeria*. These preceding studies have established the idea that when macrophages are activated with TLR and FcγRs concomitantly, they become "alternatively" activated to give rise to regulatory ones showing upregulated IL-10 production [14,29], rather than to "classically" activated ones producing inflammatory cytokines and chemokines such as IL-12 and CXCL9–11 [23,30]. Regulatory macrophages can be induced during the later stages of adaptive immune responses, in which antigen-specific IgG molecules are abundantly produced, and the main role of the regulatory macrophages seems to be limiting of the immune response and inflammation [23]. In this study, it is suggested that IL-10-producing regulatory monocytes/macrophages could be induced by employing anti-platelet IgG in place of antigen-specific antibodies.

A defect in IL-10 production could be linked to certain autoimmune and inflammatory diseases [6,11]. Therefore, in addition to understanding of the molecular mechanism that regulates the expression of this cytokine, therapeutic strategies to augment IL-10 production in inflammatory and autoimmune diseases have been attracting much attention. Our current finding that once platelets become a component of ICs with anti-integrin IgG, thereby targeting FcγRs on monocytes/platelets, could be a useful strategy for engineering IgG reagents that can interact with platelets and monocytes with suitable avidity so as to interact with these particles/cells and FcγRs, and to stimulate the IL-10-inducing pathway in monocytes. It is not known currently why IgG-platelets cannot be replaced with IgG-coated plastic microparticles in terms of converting monocytes to regulatory ones. We speculate that some undefined molecules on platelets other than integrins and FcγRIIA are necessary for efficient interaction with, and stimulation of, monocytes. Identification of such participating molecules for the platelet–monocyte interaction will enable us to develop a more efficient means of anti-inflammation than the IgG-opsonized platelets examined in this study.

The molecular nature and the immune-activating roles of FcγRIIA and other activating-type FcγRs have been extensively studied [21]. Transgenic mice expressing human FcγRIIA are highly susceptible to tissue damage by pathological antibodies in ICs, implicating FcγRIIA as a central mediator of inflammation in humans [2,31-33]. In contrast to the wealth of knowledge on FcγRIIA’s role as an inflammatory mediator, its anti-inflammatory role has not been studied sufficiently. Our present study has indicated FcγRIIA’s regulatory role in monocyte activation as well as a supporting role of this receptor on platelets, giving rise to regulatory cells via interaction with IgG-opsonized platelets. In addition to IL-10-mediated anti-inflammation, targeting to FcγRIIA and other FcγRs has also been developed to treat inflammation and cancer [21]. The latter include recombinant soluble decoy FcγRs as competitive blockers, and anti-receptor mAbs, synthetic peptides, Ig mimetics and small chemicals as FcγR blockers. Our present observations change our idea of activating FcγRs from inflammatory to regulatory receptors.

## Conclusions

Our findings of the IgG-bound platelet-induced conversion of monocytes to regulatory cells open a new avenue for the development of IL-10-inducing therapeutic strategies for inflammation, autoimmune diseases and cancer.

## Additional file

Additional file 1: Table S1. Specificities and isotypes of monoclonal IgG antibodies used for IgG-bound platelet-induced regulatory response or its blocking in this study. **Figure S1.** Platelet-reactive IgG antibodies can evoke the response. (A–C) Antibodies binding avidly to platelets can augment IL-10 release and reduce IL-1β. Using a whole PMBC preparation, the binding activity of each mAb as to platelets was assessed by flow cytometry (A). Cytokine modulation by mAbs was compared (B), and the results were summarized (C). Cytokine modulation roughly correlated with flow cytometric reactivity of mAbs to platelets. **Figure S2.** Indispensable FcRγ but not FcγRIIB, and the isotype-dependent nature of IgG-bound platelet-induced regulatory response. Bone marrow (BM)-derived cultured macrophages from wild-type (WT), FcγRIII-, FcγRIIB- or FcRγ-deficient mice were treated with an anti-human CD41 or CD42b, or anti-human CD9 mAb (isotype, IgG1) or anti-human CD36 mAb (IgG2a), and then stimulated with LPS in the presence of human platelets. The IL-10 (upper) and IL-6 (lower) levels in the culture supernatants were measured after 24 h. Data are shown as means for triplicate samples ± SEM. The results are representative of more than three independent experiments with similar results. ***P* < 0.01.

## Abbreviations

BM: Bone marrow
FcγR: Fc receptor for IgG
FcRγ: Fc receptor common γ subunit
IC: Immune complex
