# Supplementary material for: Platelets convert peripheral blood circulating monocytes to regulatory cells via immunoglobulin G and activating-type Fcγ receptors
Source: BMC Immunol. 2015 Apr 21;16:20. doi: 10.1186/s12865-015-0086-z (PMC4407389; doi:10.1186/s12865-015-0086-z)
Supplement: Additional file 1: Table S1. — Specificities and isotypes of monoclonal IgG antibodies used for IgG-bound platelet-induced regulatory response or its blocking in this study. Figure S1. Platelet-reactive IgG antibodies can evoke the response. (A–C) Antibodies binding avidly to platelets can augment IL-10 release and reduce IL-1β. Using a whole PMBC preparation, the binding activity of each mAb as to platelets was assessed by flow cytometry (A). Cytokine modulation by mAbs was compared (B), and the results were summarized (C). Cytokine modulation roughly correlated with flow cytometric reactivity of mAbs to platelets. Figure S2. Indispensable FcRγ but not FcγRIIB, and the isotype-dependent nature of IgG-bound platelet-induced regulatory response. Bone marrow (BM)-derived cultured macrophages from wild-type (WT), FcγRIII-, FcγRIIB- or FcRγ-deficient mice were treated with an anti-human CD41 or CD42b, or anti-human CD9 mAb (isotype, IgG1) or anti-human CD36 mAb (IgG2a), and then stimulated with LPS in the presence of human platelets. The IL-10 (upper) and IL-6 (lower) levels in the culture supernatants were measured after 24 h. Data are shown as means for triplicate samples ± SEM. The results are representative of more than three independent experiments with similar results. **P < 0.01. [file 12865_2015_86_MOESM1_ESM.pdf]

Supplementary Table I

Specificities and isotypes of monoclonal IgG antibodies used for IGPS or its blocking in this study

mAb	Specificity	IgG isotype
anti-CD61 (#33)	human CD61	mIgG2a
anti-CD61 (6E4)	human/mouse CD61	mIgG2a
anti-CD41 (HIP8)	human CD41	mIgG1
anti-CD42b (HIP1)	human CD42b	mIgG1
anti-CD9 (HI9a)	human CD9	mIgG1
anti-CD36 (5-271)	human CD36	mIgG2a
IV.3	human FcγRIIA/B	mIgG2b
3G8	human FcγRIIIA/B	mIgG1

Supplementary Figure S1

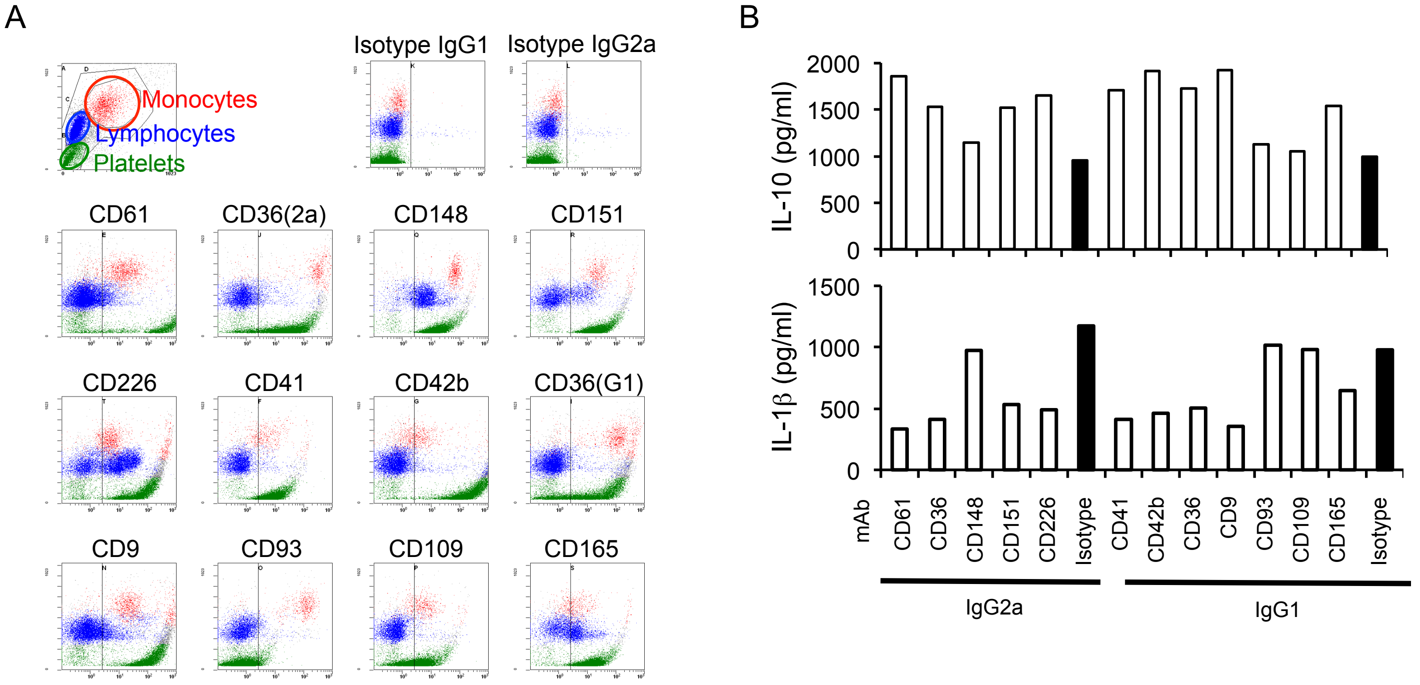

C

CD number	Alternative name or protein family	FCM reactivity Platelets[a]	Monocytes[b]	Cytokine modulation
CD41	GPIIb (Integrin αIIb)	+	+	+
CD61	GPIIIa (Integrin β3)	++	+	+
CD42b	GPIbα	++	±	+
CD36	GPIIIb/IV	++	+	+
CD9	p24, tetraspan family	++	+	+
CD93	Transmembrane protein	–	+	–
CD109	GPI-anchored protein	+	±	–
CD148	Membrane Tyr phosphatase	+	+	±
CD151	Tetraspan family protein	+	+	±
CD165	AD2	+	±	±
CD226	DNAM-1	++	+	+

[a]Flow cytometry reactivity of IgG to platelets based on results in panel C.
[b]Flow cytometry reactivity of IgG to monocytes based on results in panel C.

FIGURE S1. Platelet-reactive IgG antibodies can evoke IGPC.
(**A**–**C**) Antibodies binding avidly to platelets can augment IL-10 release and reduce IL-1β. Using a whole PMBC preparation, the binding activity of each mAb as to platelets was assessed by flow cytometry (**A**). Cytokine modulation by mAbs was compared (**B**), and the results were summarized (**C**). Cytokine modulation roughly correlated with flow cytometric reactivity of mAbs to platelets.

Supplementary Figure S2

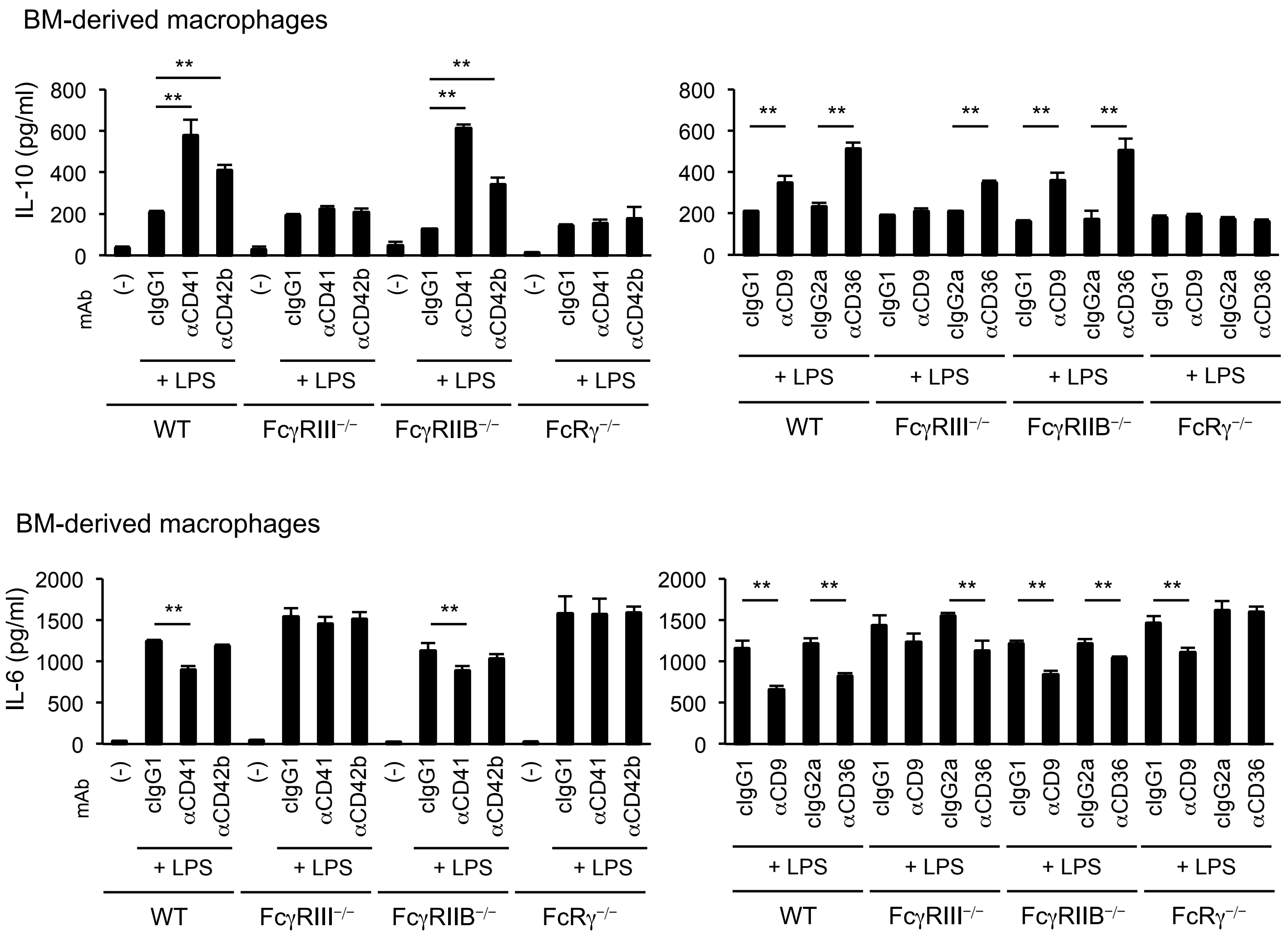

FIGURE S2. Indispensable FcRγ but not FcγRIIB, and the isotype-dependent nature of IGPC.
Bone marrow (BM)-derived cultured macrophages from wild-type (WT), FcγRIII-, FcγRIIB- or FcRγ-deficient mice were treated with an anti-human CD41 or CD42b, or anti-human CD9 mAb (isotype, IgG1) or anti-human CD36 mAb (IgG2a), and then stimulated with LPS in the presence of human platelets. The IL-10 (upper) and IL-6 (lower) levels in the culture supernatants were measured after 24 h. Data are shown as means for triplicate samples ± SEM. The results are representative of more than three independent experiments with similar results. **, $P < 0.01$.
